# Supplementary material for: Protein Quality in Infant Formulas Marketed in Brazil: Assessments on Biodigestibility, Essential Amino Acid Content and Proteins of Biological Importance
Source: Nutrients. 2021 Nov 3;13(11):3933. doi: 10.3390/nu13113933 (PMC8622549; doi:10.3390/nu13113933)
Supplement: Supplementary file 1 [file nutrients-13-03933-s001.zip › nutrients-1431959-supplementary.pdf]

Supplementary File S1

Table S1. Coded infant formulas.

Brand code	Age (mo) 0 – 6	Batch code	Age (mo) 6 - 12	Batch code
ME	1	ME1A<br>ME1B<br>ME1C	2	ME2A<br>ME2B<br>ME2C
NC	1	NC1A<br>NC1B<br>NC1C	2	NC2A<br>NC2B<br>NC2C
NN	1	NN1A<br>NN1B<br>NN1C	2	NN2A<br>NN2B<br>NN2C
DM	1	DM1A<br>DM1B<br>DM1C	2	DM2A<br>DM2B<br>DM2C
DA	1	DA1A<br>DA1B<br>DA1C	2	DA2A<br>DA2B<br>DA2C

Supplementary File S2.

Table S2. Protein label content vs. determined crude protein in different infant formulas (IFs).

Infant formulas	Protein content - experimentally evaluated		Protein label content	
	g·100 g^{-1}	g·100 kcal^{-1}	g·100 g^{-1}	g·100 kcal^{-1}
ME1A	11.61 ± 0.01^{c,*}	2.4		
ME1B	10.63 ± 0.04^{d}	2.2	9.7 g	2.0 g
ME1C	15.52 ± 0.72^{a,*}	3.2		
ME2A	14.01 ± 1.21^{b,*}	2.8		
ME2B	16.32 ± 1.44^{a,b,*}	3.3	12.0 g	2.4 g
ME2C	15.15 ± 2.01^{a,b,*}	3.1		
NC1A	9.77 ± 0.10^{d}	1.9		
NC1B	13.89 ± 0.43^{b,*}	2.7	9.3 g	1.8 g
NC1C	10.19 ± 0.66^{d}	2.0		
NC2A	13.54 ± 1.35^{b,*}	2.8		
NC2B	13.66 ± 1.25^{b,*}	2.9	9.0 g	1.9 g
NC2C	15.44 ± 0.74^{a,*}	3.2		
NN1A	11.96 ± 1.07^{c}	2.4		
NN1B	14.53 ± 1.14^{a,b,*}	2.9	11.0 g	2.2 g
NN1C	9.71 ± 1.31^{d}	2.0		
NN2A	13.21 ± 1.15^{b,c,*}	2.8		
NN2B	14.92 ± 0.74^{a,*}	3.1	11.0 g	2.3 g
NN2C	14.20 ± 0.64^{b,*}	3.0		
DM1A	15.91 ± 1.49^{a,*}	3.1		
DM1B	16.71 ± 1.38^{a,*}	3.3	11.0 g	2.2 g
DM1C	14.68 ± 2.26^{a,*}	2.9		
DM2A	15.58 ± 0.56^{a,*}	3.2		
DM2B	11.57 ± 0.43^{c,*}	2.4	14.0 g	2.9 g
DM2C	15.57 ± 0.82^{a,*}	3.2		
DA1A	11.05 ± 0.51^{d,*}	2.3		
DA1B	10.65 ± 1.02^{d}	2.2	9.8 g	2.0 g
DA1C	12.46 ± 0.31^{c,*}	2.6		
DA2A	14.54 ± 0.62^{b,*}	3.2		
DA2B	13.84 ± 1.21^{a,b,*}	3.0	11.0 g	2.4 g
DA2C	14.22 ± 1.31^{a,b,*}	3.1		

Values are expressed as the means ± SD of triplicates of each batch from the same manufacturer. Brands were designated by two capital letters, followed by number 1 or 2, indicating phase 1 or phase 2 formulas. Batches were coded as A, B or C, followed by the phase numbers. Different lowercase letters superscript within the same column indicate significant differences between infant formulas at a significance level of $p<0.01$. The symbol * within the same line indicates significant differences between experimentally determined protein contents and protein label content at a significance level ($p<0.05$) when comparing individual batch.

Supplementary File S3.

A

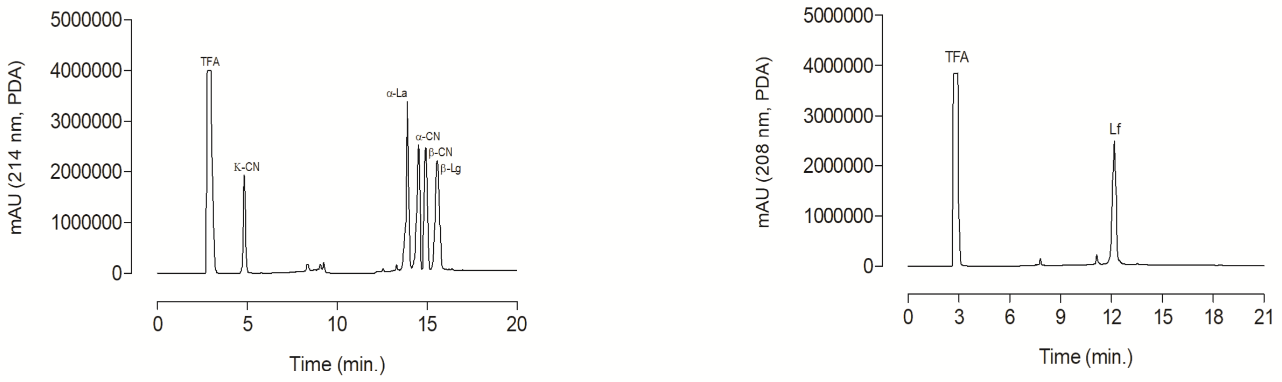

B

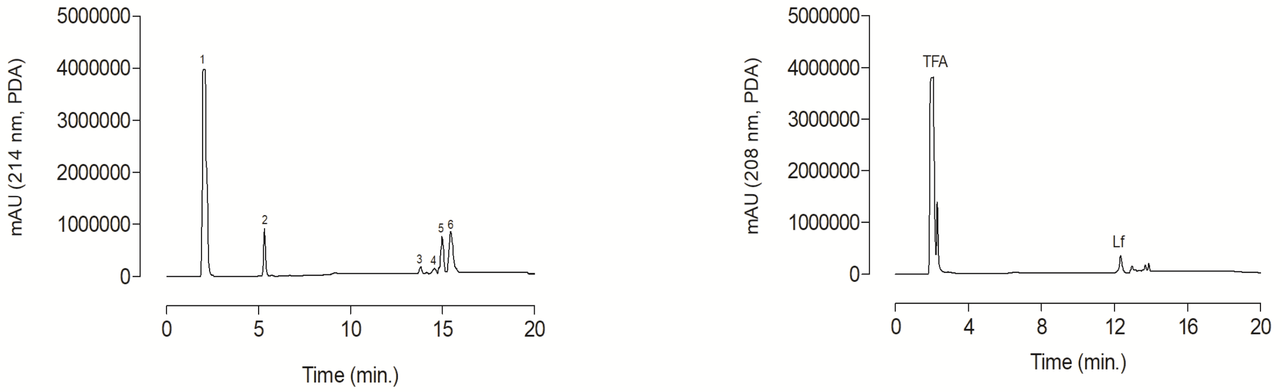

Figure S3. Representative chromatogram of a mixture of bovine milk proteins and lactoferrin standards (A) and a protein fraction chromatogram from infant formulas (**B**). Protein standards κ-CN (5 min.), α-La (14.1 min.), α-CN (13.7-14.1 min.), β-CN (14.5-14.8 min.) and β-Lg (14.8-15 min.). In figure B: 1-TFA; 2-κ-CN; 3-α-La; 4-α-CN; 5-β-CN and 6-β-Lg.

Supplementary File S4.

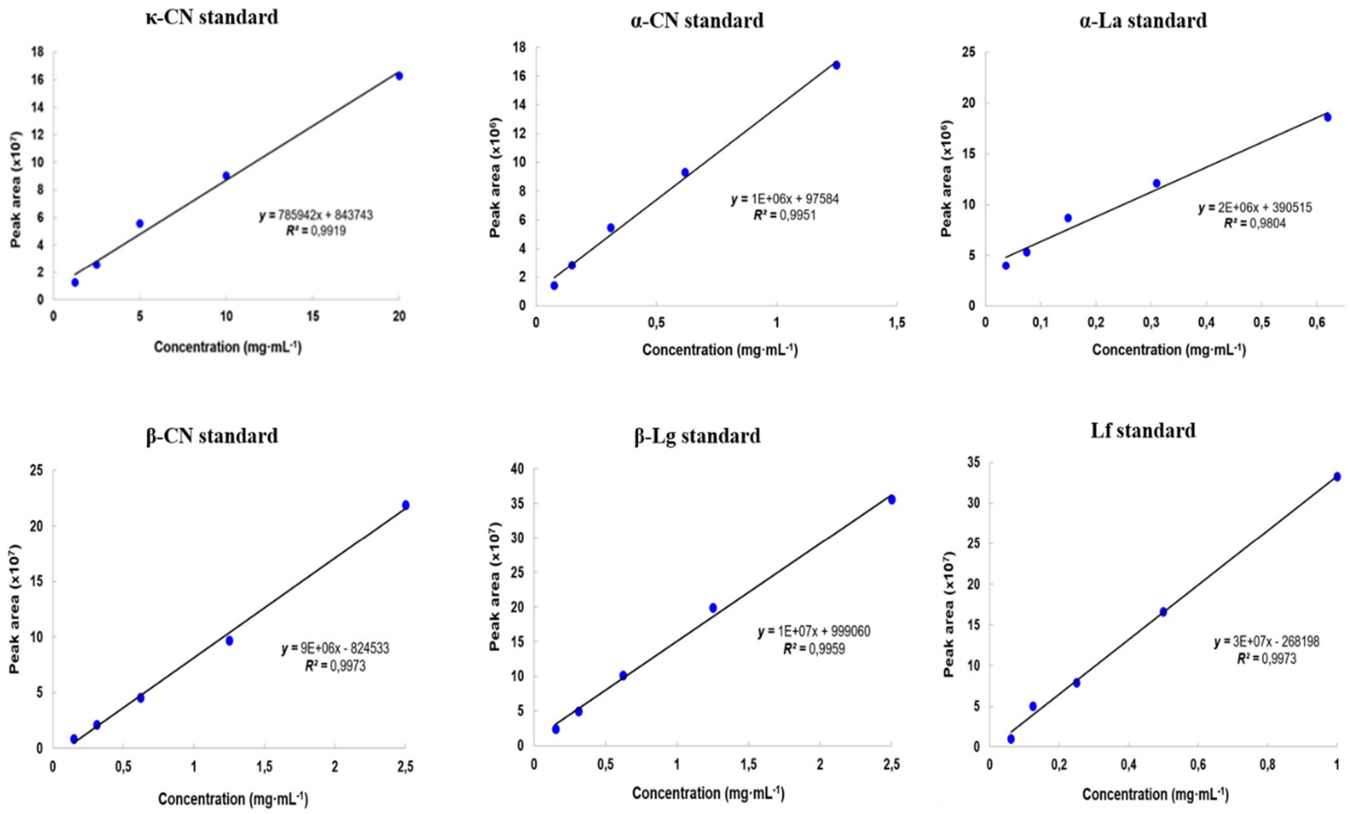

Figure S4. Standard curves of bovine milk protein fractions identified by HPLC. κ-CN (1.25 to 20 mg·mL^{-1}), α-CN (0.075 to 1.25 mg·mL^{-1}), α-LA (0.037 to 0.62 mg·.mL^{-1}), β-CN (0.15 to 2.5 mg·mL^{-1}), β-LG (0.15 to 2.5 mg·mL^{-1}) and lactoferrin (0.062 to 1.0 mg·mL^{-1}).
